# Alterations of Cerebral Perfusion and Functional Connectivity in Children With Idiopathic Generalized Epilepsy

**DOI:** 10.3389/fnins.2022.918513

**Published:** 2022-06-13

**Authors:** Guiqin Chen, Jie Hu, Haifeng Ran, Lei Nie, Wenying Tang, Xuhong Li, Qinhui Li, Yulun He, Junwei Liu, Ganjun Song, Gaoqiang Xu, Heng Liu, Tijiang Zhang

**Affiliations:** Department of Radiology, Affiliated Hospital of Zunyi Medical University, Medical Imaging Center of Guizhou Province, Zunyi, China

**Keywords:** idiopathic generalized epilepsy, cerebral blood flow, functional connectivity, arterial spin labeling, resting state fMRI

## Abstract

**Background:**

Studies have demonstrated that adults with idiopathic generalized epilepsy (IGE) have functional abnormalities; however, the neuropathological pathogenesis differs between adults and children. This study aimed to explore alterations in the cerebral blood flow (CBF) and functional connectivity (FC) to comprehensively elucidate the neuropathological mechanisms of IGE in children.

**Methods:**

We obtained arterial spin labeling (ASL) and resting state functional magnetic resonance imaging data of 28 children with IGE and 35 matched controls. We used ASL to determine differential CBF regions in children with IGE. A seed-based whole-brain FC analysis was performed for regions with significant CBF changes. The mean CBF and FC of brain areas with significant group differences was extracted, then its correlation with clinical variables in IGE group was analyzed by using Pearson correlation analysis.

**Results:**

Compared to controls, children with IGE had CBF abnormalities that were mainly observed in the right middle temporal gyrus, right middle occipital gyrus (MOG), right superior frontal gyrus (SFG), left inferior frontal gyrus (IFG), and triangular part of the left IFG (IFGtriang). We observed that the FC between the left IFGtriang and calcarine fissure (CAL) and that between the right MOG and bilateral CAL were decreased in children with IGE. The CBF in the right SFG was correlated with the age at IGE onset. FC in the left IFGtriang and left CAL was correlated with the IGE duration.

**Conclusion:**

This study found that CBF and FC were altered simultaneously in the left IFGtriang and right MOG of children with IGE. The combination of CBF and FC may provide additional information and insight regarding the pathophysiology of IGE from neuronal and vascular integration perspectives.

## Introduction

Epilepsy is a chronic disorder of the nervous system disease caused by excessive brain neuronal firing or abnormal synchronous activity that causes brain dysfunction (Falco-Walter et al., 2018). It affects more than 70 million people globally (Thijs et al., 2019). According to the 2017 seizure classification of the International League Against Epilepsy, epilepsy is divided into focal epilepsy, generalized epilepsy, and epilepsy of unknown cause. Idiopathic generalized epilepsy (IGE) refers to an epilepsy syndrome involving genetic or speculated genetic defects (Scheffer et al., 2017). Children comprise approximately 0.5 to 1% of all IGE patients (Aaberg et al., 2017). Furthermore, it has been observed that the impact of IGE on the patient’s life is more pronounced at younger ages. This may lead to abnormal brain functions in children and could place their lives at risk. The etiology, pathogenesis, clinical treatment, and prognosis of epilepsy for children differ from those for adults because the brains of children are still developing. Compared to adults, children are more likely to have epilepsy more frequently, and it is difficult to locate the epileptic foci in children. Repeated seizures can cause changes in and reconstruction of the brain functions of children, leading to cognitive dysfunction and behavioral abnormalities (Holmes, 2016; Stirling et al., 2021). Recently, neuroimaging studies, especially those that used functional magnetic resonance imaging (fMRI), including arterial spin labeling (ASL) and resting state fMRI (rs-fMRI), have become increasingly popular.

Arterial spin labeling perfusion imaging is a non-invasive MRI technique for measuring CBF without using contrast agents (Ho, 2018). Because it does not require the injection of contrast agents, it has the advantages of easy access, no radiation, short scanning times, and low costs. Recently, ASL has been used widely for the study of neurological disorders, such as epilepsy (Schertz et al., 2020), Alzheimer’s disease (Iturria-Medina et al., 2016; Benedictus et al., 2017), Parkinson’s disease (Lin et al., 2017), and preoperative brain tumor grading (Shen et al., 2016; Ningning et al., 2017). Furthermore, ASL is capable of explaining the neural basis of IGE and enabling early diagnosis. Some previous studies based on ASL have shown that IGE patients have insufficient cerebral perfusion, mainly in the thalamus, superior middle brain, and left cerebellum (Sone et al., 2017). Other studies have shown increased cerebral perfusion mainly in the left parahippocampal gyrus, left middle temporal gyrus, and left fusiform gyrus (Chen et al., 2016). However, because of differences in image acquisition, samples, and analysis methods, detailed results across studies have been inconsistent.

With rs-fMRI, changes in blood oxygen level-dependent (BOLD) signals are detected. These can be used to study the spontaneous neural activity of the brain in the resting state and to evaluate the FC and networks of various brain regions. Furthermore, because rs-fMRI does not involve the need to perform specific tasks, participants can cooperate well. It has been used in the field of neuropsychiatry, where it is especially suitable for studying neuropsychiatric diseases of patients with cognitive and attentional disorders, or the inability to cooperate, including bipolar disorder (Bellani et al., 2020), psychosis (Collin et al., 2020), Alzheimer’s disease, and mild cognitive impairment (Ibrahim et al., 2021). Based on the region of interest (ROI) analysis method, Luo et al. (2011) studied the default mode network (DMN) of patients with absence epilepsy and observed that the functional connections of the frontal, parietal, and temporal lobes were significantly reduced. Kim et al. (2014) found a decrease in FC was found in both the precuneus cortex and medial prefrontal cortex of adult IGE patients. Based on the independent component analysis method, Li et al. (2015) showed FC changes in the dorsal attention network, salience network, and DMN in children with IGE-associated and dementia.

Based on these previous studies, CBF and FC are abnormal in IGE patients. However, the neurobiological mechanism of IGE in children is unclear, and there are no effective treatments available. Moreover, the correlation between CBF and internal brain function changes is unclear. Recently, Liang et al. (2013) combined ASL and rs-fMRI and found a close relationship between CBF and brain FC in the default network and executive control network of healthy adults. Tan et al. (2020) combined ASL and rs-fMRI and observed abnormal alterations in left amygdala perfusion and FC in patients with attention deficit/hyperactivity disorder; they also reported that neuronal and vascular perspectives of attention deficit/hyperactivity disorder will provide a deeper understanding of its pathophysiology. Joint ASL and rs-fMRI studies of patients with disorders of the nervous system, such as Alzheimer’s disease (Zheng et al., 2019), schizophrenia (Zhu et al., 2017), and depression (He et al., 2019), have been performed. Therefore, we speculated that areas showing CBF abnormalities and brain dysfunction in IGE patients may overlap. However, the simultaneous alteration of CBF and FC in IGE patients remains largely unknown. Therefore, this study aimed to explore cerebral CBF and FC in children with IGE by combining ASL with rs-fMRI and analyzing the correlations between CBF and FC changes and clinical variables to help elucidate the pathophysiological mechanism of IGE in children.

## Materials and Methods

### Subjects

This study involved sixty-three subjects. There were 28 children with IGE and 35 healthy controls (HCs). The study protocol was approved and was performed in accordance with the recommendations of the Medical Research Ethics Committee of Zunyi Medical University’s Affiliated Hospital. Written informed consent was obtained from all participants in accordance with the Declaration of Helsinki. Children with IGE underwent a complete neuropsychological assessment using the Chinese version of the Wechsler Intelligence Scale for Children, including verbal, performance, and full-scale intelligence quotient (IQ) assessments, on the same day as the MRI scan.

Idiopathic generalized epilepsy patients were required to fulfill the following inclusion criteria: (I) diagnosis of IGE according to the 2010 International League Against Epilepsy diagnostic criteria (II) underwent a normal routine MRI examination of the brain (III) and age 6–16 years. The exclusion criteria were as follows: (I) other neuropsychiatric diseases, (II) history of drug abuse, (III) craniocerebral trauma or surgical history, (IV) intracranial organic lesions or serious artifacts; and (V) head motion exceeding ± 2 mm or ± 2°.

The following were inclusion criteria for the HCs: (I) underwent a normal routine MRI examination of the brain and (II) age 6–16 years. The exclusion criteria were as follows: (I) other neurological/psychiatric conditions, (II) history of drug abuse, (III) cognitive and memory disorders, and (IV) head motion exceeding ± 2 mm or ± 2.

### Data Acquisition

A 3.0-T HDxt system (GE Healthcare, Milwaukee, WI, United States) was used to acquire all MRI results. ASL perfusion imaging was performed using pseudocontinuous labeling with three-dimensional fast spin-echo acquisition and background suppression. The following parameters were used: repetition time, 4,599 ms; echo time, 9.8 ms; post-label delay, 1,525 ms; spiral-in readout of eight arms with 512 sample points; field of view, 240 mm × 240 mm; slice number, 36; and slice thickness, 4 mm. The rs-fMRI data were acquired using an echo planar imaging sequence with the following parameters: repetition time, 2,000 ms; echo time, 30 ms; field of view, 240 mm × 240 mm; flip angle, 90°; slice number, 33; slice thickness, 4 mm; and volume, 210. The participants were instructed to keep their eyes closed, relax, remain still, think of nothing, and not fall asleep during the scanning process.

### Rs-fMRI Data Preprocessing

Preprocessing of the MRI data was performed with Data Processing Assistant for Resting-State Brain Imaging (DPARSF) (Chao-Gan and Yu-Feng, 2010). First, the first 10 parts of each participant’s recording were discarded, and the remaining parts were corrected for temporal differences and head motion. Next, functional data were realigned with echo planar imaging templates of the Montreal Neurological Institute (MNI). Standard spaces were spatially normalized using non-linear registration and then resampled to 3-mm isotropic voxels. Spatial smoothing was performed with a 6-mm full-width at half-maximum Gaussian kernel, followed by linear detrending of the time series. Thereafter, a regressing procedure was performed for the nuisance covariates derived from Friston’s 24 head movement estimates, white matter signals, and cerebrospinal fluid signals. Finally, functional images were temporally bandpass-filtered (0.01–0.1 Hz).

### Cerebral Blood Flow Data Preprocessing and Statistical Analysis

Cerebral blood flow images were obtained from the original ASL data using an MRI scanner post-processing workstation. We acquired CBF images and processed the data using SPM12^1^. The CBF images of each patient’s structural space were transformed to the Montreal Neurological Institute standard space. Subsequently, we calculated a z-score from the spatially normalized CBF images of each participant by subtracting the mean and dividing by the standard deviation of all voxels across all subjects. Finally, all CBF images were smoothed using a 6-mm × 6-mm × 6-mm Gaussian kernel filter with full-width at half-maximum. Further statistical analyses using an independent two-sample *t* test were performed to compare the groups using age and sex as covariates. The significance threshold was set using the Gaussian random field (GFR) correction (*P* < 0.05). Based on Pearson’s correlation analysis, abnormal CBF was correlated with IQ, disease course, and age at onset. The significance level was set at *P* < 0.05.

### Functional Connectivity and Statistical Analysis

A seed-based FC analysis was conducted. First, based on the CBF results, four regions with significant differences between groups were selected as masks and defined as ROIs during the FC analysis. Thereafter, using an independent two-sample *t* test with age and sex as covariates, we compared the two groups (IGE patients vs. HCs). The significance threshold was set using the GFR correction (*P* < 0.05, two-tailed). Finally, we extracted the abnormal FC of brain regions. Pearson’s correlation analysis was performed for brain regions with abnormal FC in children with IGE and IQ, disease course, and age at onset. The significance level was set at *P* < 0.05.

## Results

### Clinical Data

Demographic and clinical characteristics of the 28 children with IGE (14 females and 14 males; age, 11.71 ± 2.89 years) and 35 HCs (19 females and 16 males; age, 11.88 ± 2.48 years) are described in Table 1. There were no significant differences in sex (*P* = 0.958, *P* > 0.05, chi-square test), age (*P* = 0.801, *P* > 0.05, two-sample *t*-test), and education (*P* = 0.385, *P* > 0.05, two-sample *t*-test) between the IGE patients and HCs.

**TABLE 1:** Demographic and clinical information.

	IGE group	Healthy control group	*t/*χ^2^	*P* value
Number	28	35	–	–
Age (years)	11.71 ± 2.89	11.88 ± 2.48	−0.253*	0.801*
Males/Females	14/14	19/16	0.003^#^	0.958^#^
Education (years)	6.21 ± 2.67	6.85 ± 2.03	−0.881*	0.385*
Age of onset (years) (years)	7.45 ± 3.78	–	–	–
Duration (years)	4.05 ± 3.83	–	–	–
VIQ	90.07 ± 20.08	–	–	–
PIQ quotient	84.00 ± 16.76	–	–	–
FIQ	86.53 ± 18.26	–	–	–

*Two-sample t-test; ^#^Chi-square test; IGE, idiopathic generalized epilepsy; FIQ, full-scale intelligence quotient; PIQ, performance intelligence quotient; VIQ, verbal intelligence quotient.

### Spatial Distribution of Cerebral Blood Flow

**FIGURE 1:**
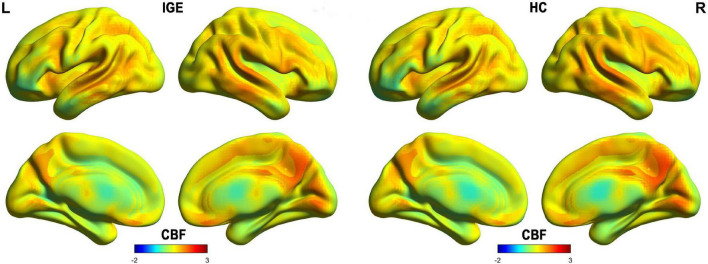
Spatial distribution maps of CBF at the group level. For each group (i.e., IGE or HC), the individual CBF maps were normalized to z-scores and then averaged across subjects to generate a group-level map. IGE, idiopathic generalized epilepsy; CBF, cerebral blood flow; HC, healthy controls.

The spatial distribution map of CBF in the HCs and IGE patients are shown in Figure 1. Subtle differences were observed between these groups (IGE patients: 53.28 ± 8.09 ml/100 g/min; HCs: 53.85 ± 7.60 ml/100 g/min; two-sample *t* test; *t* = 0.29; *P* = 0.773) in the whole brain matter. Increased blood flow was detected mainly in the left inferior frontal gyrus (IFG), middle frontal gyrus, cingulate gyrus, bilateral middle temporal gyrus (MTG), thalamus, right middle frontal gyrus (MOG), and right medial prefrontal cortex.

### Cerebral Blood Flow and Functional Connectivity Changes in Idiopathic Generalized Epilepsy Patients

**FIGURE 2:**
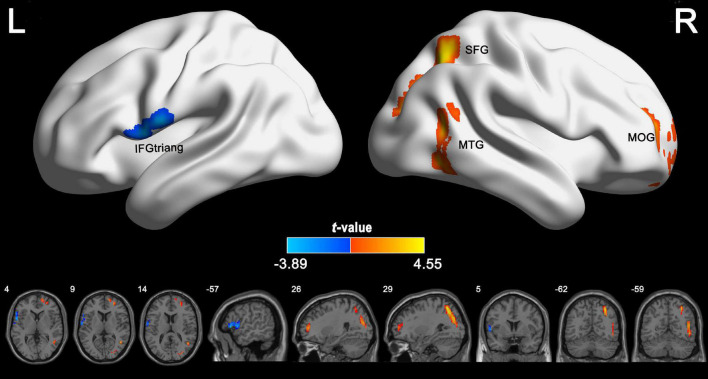
Group differences in CBF between IGE patients and healthy controls. The independent two-sample *t* test was conducted between the IGE group and the healthy control group. All results were corrected for multiple comparisons (GRF-corrected, *P* < 0.05). The cold colors denote significantly decreased CBF in the IGE patients. The warm colors denote significantly increased CBF in the IGE patients. CBF, cerebral blood flow; GRF, Gaussian random field; IGE, idiopathic generalized epilepsy.

**FIGURE 3:**
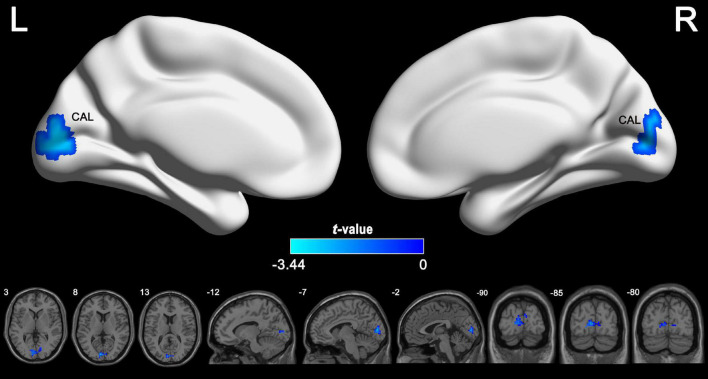
Between-group comparisons for the seed-based functional connectivity with the seeds of left IFGtriang. The independent two-sample *t* test was conducted between the IGE group and HC. All results were corrected for multiple comparisons (GRF, *P* < 0.05). CBF, cerebral blood flow; HC, healthy controls; GRF, Gaussian random field; IGE, idiopathic generalized epilepsy. IFGtriang, inferior frontal gyrus, triangular part.

**FIGURE 4:**
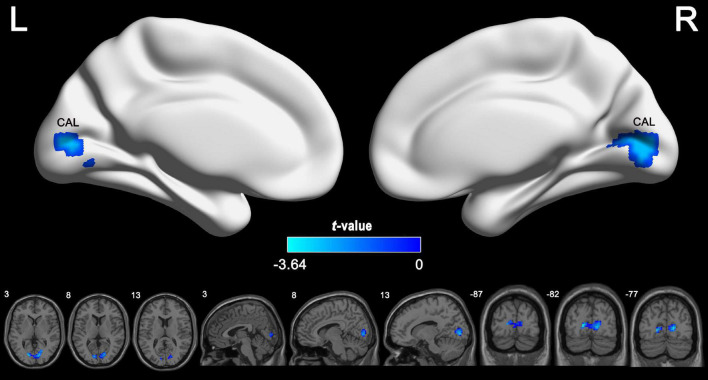
Between-group comparisons for the seed-based functional connectivity with the seeds of right MOG. The independent two-sample *t* test was conducted between the IGE group and HC. All results were corrected for multiple comparisons (GRF, *P* < 0.05). CBF, cerebral blood flow; HC, healthy controls; GRF, Gaussian random field; IGE, idiopathic generalized epilepsy; MOG, middle occipital gyrus.

According to the voxel-wise ASL analysis, decreased CBF in IGE patients was mainly present in the triangular part of the left IFG (IFGtriang) and increased CBF in IGE patients was mainly present in the right MTG, MOG, and superior FG (SFG) (Figure 2 and Table 2). By identifying the four brain regions, we were able to explore the whole brain FC of each region. We conducted two-sample *t*-tests to examine the differences in FC of the regions of interest in the IGE patients and HCs. Compared with the HCs, we found there was a significant decrease in connectivity in the left IFGtriang with the CAL (Figure 3), and that the FC between the right MOG and bilateral CAL was decreased in children with IGE (Figure 4).

**TABLE 2:** Brain regions with significant differences in CBF between IGE and HC groups.

Voxels, n	MNI coordinates, mm			Brain regions (AAL)	Peak *t* values
		(x, y, z)			
87	48	−55	−5	right MTG	4.2175
117	15	56	−5	right SFG	4.1687
91	−57	23	1	left IFGtriang	−3.8892
170	33	−70	34	right MOG	4.5459

*The significance threshold of the datas was set to GRF correction (P < 0.05, two tailed) with age, sex as covariates. CBF, cerebral blood flow; MTG, middle temporal gyrus; MOG, middle occipital gyrus; SFG, superior frontal gyrus; IFGtriang, inferior frontal gyrus, triangular part.*

### Relationship Between Cerebral Blood Flow and Functional Connectivity Changes and Clinical Variables

**FIGURE 5:**
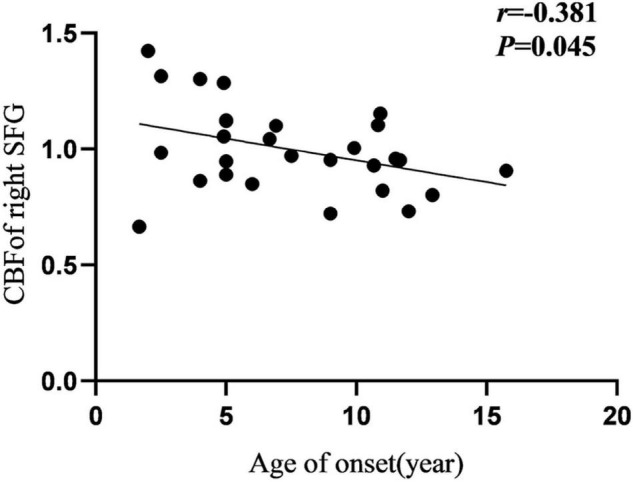
Pearson correlation between CBF of right SFG and age of onset in IGE patients. CBF, cerebral blood flow; SFG, superior frontal gyrus.

**FIGURE 6:**
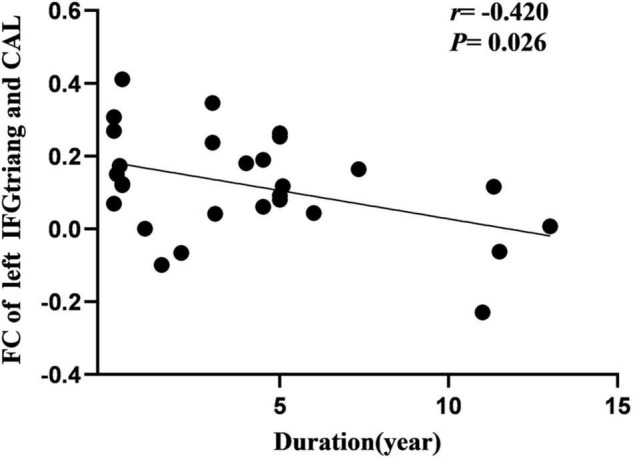
Pearson correlation between FC of left CAL and course of disease in IGE patients. FC, functional connectivity; CAL, calcarine fissure.

Cerebral blood flow of the right SFG was negatively correlated with the age at onset in the IGE group (Figure 5). Additionally, FC of the left IFGtriang and ipsilateral CAL were negatively correlated with IGE duration (Figure 6). However, no significant correlations were observed between the intelligence scale and CBF or FC.

## Discussion

This study combined three-dimensional ASL perfusion with rs-fMRI to examine changes in CBF and potential FC disruptions in CBF-altered regions in children with IGE. Compared with HCs, children with IGE had significant abnormal perfusion in the right MOG and left IFGtriang. A seed-based FC analysis was used to evaluate the potential changes in these abnormal regions and found simultaneous decreases in the FC between the left IFGtriang and CAL and between the right MOG and bilateral CAL, which could help comprehensively elucidate the neuropathological mechanism of IGE in children.

### Cerebral Blood Flow Changes in Idiopathic Generalized Epilepsy Patients

Previous studies have consistently reported significant abnormal perfusion in the frontal, temporal, and occipital lobes of IGE patients. The regions showing perfusion abnormalities in the frontotemporal lobe also showed structural and functional abnormalities similar to those reported by other MRI studies (Ke et al., 2017; Deng et al., 2019; Li et al., 2020). In this study, hypoperfusion was found in the left IFGtriang. This is partially consistent with the findings reported by Joo et al. (2006), who used single-photon emission computed tomography to study CBF in the IGE group and observed that multiple brain regions, including the IFG, showed low perfusion. Moreover, we observed reduced CBF in the frontal lobe, indicating dysfunction of the nerves and blood vessels in that area. Pathological processes with IGE can be linked to neurovascular dysfunction, especially CBF abnormalities. More specifically, the stability of neuronal cells is reduced in patients with epilepsy. When epilepsy occurs, brain ischemia and hypoxia are aggravated, resulting in failure of the sodium pump. An increase in Na^+^ influx leads to depolarization, causing vascular neuronal damage to a certain extent. In particular, when the endothelial function of the local capillaries is damaged, local CBF is reduced (Barth and Mody, 2011; Gooshe et al., 2015; Tewolde et al., 2015). Therefore, we can speculate that the IFGtriang is vascularly dysfunctional, and that decreases in CBF lead to the destruction of the vascular barrier, glial hyperplasia, and inflammation and induce excessive excitement of neuronal networks, thereby promoting epilepsy and leading to reduced perfusion. Combined with a neurobiological analysis, this speculation may help explain CBF abnormalities in children with IGE more comprehensively.

There have been reports of increased CBF during the postictal phase of typical absence seizures and the interictal phase of myoclonic epilepsy in adolescents (Nehlig et al., 2004; Tae et al., 2007). In another study, CBF increased in the left MTG, left parahippocampal gyrus, and left fusiform gyrus of IGE patients, and a negative correlation between age at onset and prolongation of arterial transit time of the left superior temporal gyrus was observed. That study showed that IGE patients may have cortical dysfunction in the temporal lobe and fusiform gyrus, which may be associated with epileptic activity (Chen et al., 2016). During our study, children with IGE had higher regional CBF in the right MTG and SFG regions, which is highly consistent with previously reported findings (Tae et al., 2007; Chen et al., 2016). Most epilepsy occurs in the frontotemporal lobe, and microstructural changes in these regions have been observed in IGE patients (Betting et al., 2010; Zhang et al., 2016). Another study revealed that epileptiform discharges in patients with juvenile myoclonic epilepsy are not generalized with bilateral synchronous diffusion onset. Additionally, during propagation, discharges have localized onset and a restricted cortical network including regions of the frontal cortex and temporal cortex (Holmes et al., 2010). Previous studies demonstrated that the involvement of leukocyte-endothelial interactions in epilepsy is inextricably linked to hyperperfusion-induced seizures (Fabene et al., 2008; Storti et al., 2014). Our results and the aforementioned evidence show that IGE patients have a pattern of high perfusion, and that abnormal CBF perfusion in the MTG and SFG may be associated with epileptiform activity in IGE patients.

The MOG is the visual sensory cortex, which is mainly responsible for processing visual information (Müller and Kleinschmidt, 2003). We observed an increase in CBF in the right MOG and speculated that this may be related to visual abnormalities before the attack, such as blurred vision and deformed visual changes, or to damage to the visual cortex, indicating abnormalities in the occipital network involved in the neurobiological mechanism of IGE, in some patients. However, this increase in CBF in the right MOG was inconsistent with previous results and may be related to the different sample sizes, IGE subtypes, and medications used during this study.

### A Seed-Based Functional Connectivity Analysis of Idiopathic Generalized Epilepsy Patients

During the seed-based FC analysis, we found a significant decrease in the FC in the left IFGtriang with the CAL, and the FC between the right MOG and bilateral CAL was decreased in children with IGE.

Previous studies have reported abnormal FC of the frontal lobe with other regions, such as the thalamus, posterior cingulate gyrus, and orbitofrontal cortex in IGE patients (Hamandi et al., 2006; Bai et al., 2011; Kim et al., 2014). During our study, we found that the FC between the left IFGtriang and ipsilateral CAL was reduced. The IFGtriang area is a part of the prefrontal cortex that has a key role in executive functions such as attention and executive ability. The CAL is involved in visual information processing, and we speculated that cerebral perfusion of the frontal lobe was closely related to the FC of the ipsilateral CAL, suggesting that children with IGE might have visual impairment (i.e., abnormal frontal perfusion in children with IGE might be accompanied by abnormal visual function).

The MOG and CAL are important components of the visual sensory cortex and visual network, and they have a critical role in the processing of visual information. Salinas and Szabó (2015) observed that FC between the occipital cortex and multiple brain areas of the epilepsy network in the IGE group was significantly abnormal. Similarly, Szabó et al. (2007) observed that photosensitive baboons with IGE had frequent discharges in the parietal cortex and occipital cortex during seizures and interictal periods. Combined with the results of this study, the decrease in FC between the occipital lobe and CAL in children with IGE may be related to the photosensitivity (Ákos Szabó et al., 2016) of some patients with epilepsy, suggesting the presence of FC abnormality in the visual cortex network, which may be a potential cause of impaired visual function.

During our study, we found that areas with perfusion abnormalities in children with IGE had alterations in FC. The BOLD signal depends on the concentration of deoxyhemoglobin in the blood and is regulated by changes in the CBF and cerebral blood volume (Driver et al., 2017) and abnormal perfusion changes in the concentration of local deoxyhemoglobin in the brain, leading to changes in BOLD signal intensity and whole brain FC. Therefore, abnormal perfusion with IGE may lead to changes in brain FC.

### Relationship Between Cerebral Blood Flow and Functional Connectivity Changes and Clinical Variables

During this study, we observed that CBF in the right SFG was negatively correlated with the age at IGE onset, signifying that a younger age at onset is associated with a greater CBF increase. The frontal lobe is the advanced center of brain development and embodiment of human wisdom. It is closely related to attention, cognition, and executive control function. Therefore, it can be proposed that CBF changes in the SFG may hurt the ability of the region to recruit when responding to task demands, and thus may cause impaired cognitive functioning and attention disturbances. Furthermore, between abnormal FC of the left IFGtriang and CAL was negatively correlated with IGE duration. The frontal lobe and CAL have important roles in cognitive and visual processing. Previous studies have found that changes in the prefrontal function correlate well with disease duration. Kay et al. (2013) performed fMRI studies of IGE and observed that there was a decrease in FC between the frontal lobe and posterior cingulate gyrus, and that FC was negatively correlated with disease duration. Using rs-fMRI, McGill (McGill et al., 2012) observed that the FC of the prefrontal cortex of the DMN was significantly negatively correlated with the duration of epilepsy, suggesting that DMN connectivity decreased with the prolonged duration of epilepsy. During this study, the FC between the IFGtriang and CAL decreased and was negatively correlated with the duration of the disease, suggesting that FC changes between the IFGtriang and CAL may indicate IGE disease progression, thus reflecting the chronic damage to the brain function network caused by epilepsy and the development of the brain functional networks in children with IGE.

### Limitations

There were some limitations to this study. First, intelligence of the HCs was not evaluated; however, they attended normal primary and secondary schools. The performance of the HCs during the MRI scanning sessions and at school was to be normal. Second, there were no data regarding the IGE subtypes. Third, previous studies showed that antiepileptic drugs may affect the results of functional MRI. Jansen et al. (2006) reported that the activation of language-mediated regions in the prefrontal cortex of epilepsy patients treated with topiramate was significantly lower than that of the untreated group. Moeller et al. (2008) found no signal changes in the striatum-thalamus-cortex in unmedicated children with absence epilepsy; however, signal changes in that region were found in the medication group. The children with IGE in this study were treated with antiepileptic drugs. Unfortunately, we did not collect enough data to investigate the effects of these drugs on IGE neural activity. Therefore, the findings of this study need to be interpreted carefully. In the future, we will attempt to design a study with stricter criteria and data about IGE subtypes and drugs to perform further analyses.

## Conclusion

We observed simultaneous changes in the CBF and FC in the left IFGtriang and right MOG in children with IGE. The combination of ASL and rs-fMRI and the integration of neuronal and vascular information can add to our understanding of the pathophysiology of children with IGE.

## Data Availability Statement

The original contributions presented in this study are included in the article/supplementary material, further inquiries can be directed to the corresponding authors.

## Ethics Statement

The studies involving human participants were reviewed and approved by the Medical Ethics Committee of Affiliated Hospital of Zunyi Medical University. Written informed consent to participate in this study was provided by the participants’ legal guardian/next of kin.

## Author Contributions

GC and JH contributed to the conception, design, and radiological expertise, helped to select and assess cases, and drafted manuscript. JL, GS, and GX contributed radiological expertise and carried out the statistical analysis and designed the study. HR and LN contributed to the drafting and revision of the manuscript. WT, QL, XL, and YH offered data collection. TZ and HL reviewed and critiqued the manuscript and approved the final manuscript. All authors have read and approved the final manuscript.

## Conflict of Interest

The authors declare that the research was conducted in the absence of any commercial or financial relationships that could be construed as a potential conflict of interest.

## Publisher’s Note

All claims expressed in this article are solely those of the authors and do not necessarily represent those of their affiliated organizations, or those of the publisher, the editors and the reviewers. Any product that may be evaluated in this article, or claim that may be made by its manufacturer, is not guaranteed or endorsed by the publisher.

## Footnotes

1
http://www.fil.ion.ucl.ac.uk/spm
