# Corrigendum: Alterations of cerebral perfusion and functional connectivity in children with idiopathic generalized epilepsy

**DOI:** 10.3389/fnins.2022.1011310

**Published:** 2022-08-23

**Authors:** Guiqin Chen, Jie Hu, Haifeng Ran, Lei Nie, Wenying Tang, Xuhong Li, Qinhui Li, Yulun He, Junwei Liu, Ganjun Song, Gaoqiang Xu, Heng Liu, Tijiang Zhang

**Affiliations:** Department of Radiology, Affiliated Hospital of Zunyi Medical University, Medical Imaging Center of Guizhou Province, Zunyi, China

**Keywords:** idiopathic generalized epilepsy, cerebral blood flow, functional connectivity, arterial spin labeling, resting state fMRI

In the published article, there was an error in Figure 2 as published. In Figure 2, the annotations of SFG and MOG in the brain region were misplaced. The corrected Figure 2 appears below.

**Figure 2:**
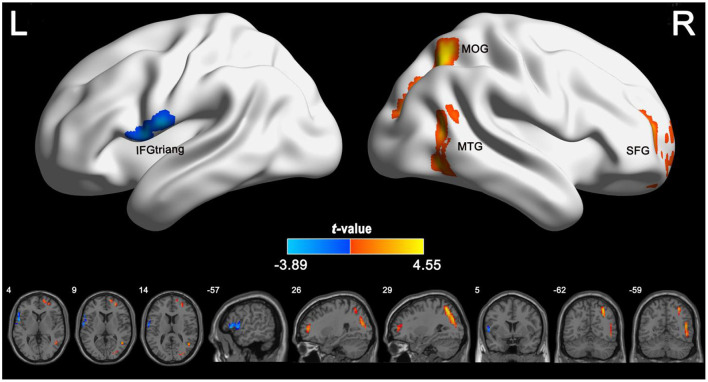
Group differences in CBF between IGE patients and healthy controls. The independent two-sample *t* test was conducted between the IGE group and the healthy control group. All results were corrected for multiple comparisons (GRF-corrected, *P* <0.05). The cold colors denote significantly decreased CBF in the IGE patients. The warm colors denote significantly increased CBF in the IGE patients. CBF, cerebral blood flow; GRF, Gaussian random field; IGE, idiopathic generalized epilepsy.

The authors apologize for this error and state that this does not change the scientific conclusions of the article in any way. The original article has been updated.

## Publisher's note

All claims expressed in this article are solely those of the authors and do not necessarily represent those of their affiliated organizations, or those of the publisher, the editors and the reviewers. Any product that may be evaluated in this article, or claim that may be made by its manufacturer, is not guaranteed or endorsed by the publisher.

